# Environmental Cues Modulate Microglial Cell Behavior Upon Shiga Toxin 2 From Enterohemorrhagic *Escherichia coli* Exposure

**DOI:** 10.3389/fcimb.2019.00442

**Published:** 2020-01-08

**Authors:** Clara Berdasco, Maite Duhalde Vega, María Victoria Rosato-Siri, Jorge Goldstein

**Affiliations:** ^1^Laboratorio de Neurofisiopatología, Consejo Nacional de Investigaciones Científicas y Técnicas, Facultad de Medicina, Instituto de Fisiología y Biofísica “Houssay”, Universidad de Buenos Aires, Buenos Aires, Argentina; ^2^Instituto de Química y Fisicoquímica Biológicas, Universidad de Buenos Aires, CONICET, Buenos Aires, Argentina

**Keywords:** Shiga Toxin 2, microglial cell primary cultures, LPS challenge, heat shock exposure, receptor Gb3-pathway

## Abstract

Shiga toxin (Stx) produced by enterohemorrhagic *E. coli* produces hemolytic uremic syndrome and encephalopathies in patients, which can lead to either reversible or permanent neurological abnormalities, or even fatal cases depending on the degree of intoxication. It has been observed that the inflammatory component plays a decisive role in the severity of the disease. Therefore, the objective of this work was to evaluate the behavior of microglial cell primary cultures upon Stx2 exposure and heat shock or lipopolysaccharide challenges, as cues which modulate cellular environments, mimicking fever and inflammation states, respectively. In these contexts, activated microglial cells incorporated Stx2, increased their metabolism, phagocytic capacity, and pro-inflammatory profile. Stx2 uptake was associated to receptor globotriaosylceramide (Gb3)-pathway. Gb3 had three clearly distinguishable distribution patterns which varied according to different contexts. In addition, toxin uptake exhibited both a Gb3-dependent and a Gb3-independent binding depending on those contexts. Altogether, these results suggest a fundamental role for microglial cells in pro-inflammatory processes in encephalopathies due to Stx2 intoxication and highlight the impact of environmental cues.

## Introduction

Shiga toxin-producing *Escherichia coli* (STEC) causes hemorrhagic colitis, and hemolytic uremic syndrome (HUS) once the toxin enters circulation from the gut (Karmali, 2004). HUS is an orally acquired infective illness produced by the ingestion of contaminated food, water and/or cross infection, and includes thrombocytopenia, microangiopathic hemolytic anemia, and acute renal failure (Gianantonio et al., 1973). In addition, Shiga toxin 2 (Stx2) targets other organs like the brain, inducing encephalopathies (Obata, 2010). Neurological damage produced by Stx2 (Ashkenazi et al., 1994; Siegler, 1994) has gained notoriety in Argentina and throughout the world. A multicenter, observational, retrospective, and cross-sectional study recently conducted by the National Epidemiological Surveillance System of Argentina concluded that central nervous system (CNS) involvement by STEC was the main predictor of death in patients with HUS (Alconcher et al., 2018).

STEC may produce two variants of Shiga toxin, Shiga toxin type 1 (Stx1) and/or Shiga toxin type 2 (Stx2); both have the same mode of action but they are antigenically different (Melton-Celsa, 2014). Stx2, the endemic variant that predominates in Argentina, is a protein formed by a catalytic subunit A (StxA) and five subunits B (StxB) related with toxin binding. StxA possesses N-glycosidase activity and inhibits protein biosynthesis. To perform this task it must be transported to the cytosol by StxB (Johannes and Decaudin, 2005; Sandvig and van Deurs, 2005) through its receptor, located in the cell membrane. Globotriaosylceramide (Gb3) is a glycosphingolipid expressed on the cell membrane of some mammalian cells and it was described to be involved in cellular signaling. In addition, Gb3 has been identified as a primary receptor for various toxins including Stx1 and Stx2 (Bekri et al., 2006). Gb3 may serve as a precursor for the synthesis of more complex globo-series glycosphingolipids, such as globotetraosylceramide (Gb4) (Kavaliauskiene et al., 2017).

It has been observed that Stx2 intracerebroventricular-administration in rat brains exerts its neurotoxic effect through its Gb3 receptor in post-synaptic neurons (Tironi-Farinati et al., 2010). Indeed, neuronal degeneration and astrocytic reaction were found in several regions of the brain (Boccoli et al., 2008).

An inflammatory component of HUS in the brain was postulated through the observation that damage to the neurovascular component could be attenuated by the administration of dexamethasone, an anti-inflammatory drug (Pinto et al., 2013). These results were in agreement with previous studies by other groups in endothelial cells cultures which showed the contribution of pro-inflammatory lipopolysaccharide (LPS) to cytotoxicity upon Shiga toxins exposure (Louise and Obrig, 1992).

Microglial (MG) cells can be postulated as a central target in the harmful action caused by Stx2, as they belong to the monocyte-macrophage immune cell lineage (Xing et al., 2011). Along the same lines, our group has recently demonstrated in a translational murine model of HUS-derived encephalopathy that systemic sub lethal Stx2 induces MG cell reactivity in the striatum and the hippocampus (Pinto et al., 2018; Berdasco et al., 2019). We hypothesized that MG cells might play a pivotal role in the inflammatory effects of Stx2 observed in the brain and, thus, define the severity of encephalopathies in patients. This state of affairs prompted us to hypothesize that Stx2, either the holotoxin or the Stx2B subunits, exerted a direct biological effect on MG cell primary cultures. Therefore, functional parameters, such as MG cell activation, cytology, metabolism, cytokine expression levels, and phagocytic status were assayed using heat shock exposure and LPS challenge to determine whether culture conditions affect MG cell sensitivity and responsiveness. The present work demonstrates that MG cells exhibit both a Gb3-independent and Gb3-cannonical pathway for Stx2 uptake. Altogether, the present results suggest a fundamental role of MG cells in the pro-inflammatory processes underlying encephalopathies due to STEC exposure.

## Materials and Methods

### Ethics Statement

All experimental procedures were performed in accordance with the guidelines of the Institutional Review Board at Buenos Aires University Council of Animal Care and were in accordance with the National Institutes of Health Guide for the Care and Use of Laboratory Animals. Animal protocols were approved by the School of Medicine Committee on Ethics and Animal Research (CICUAL Number: 046/2017).

### Animals

Wistar rats were housed under controlled temperature (22 ± 2°C) in an artificially lit animal room under a 12 h-light/dark cycle and fed water and food *ad libitum*.

### MG Cell Primary Cultures

**Figure 1:**
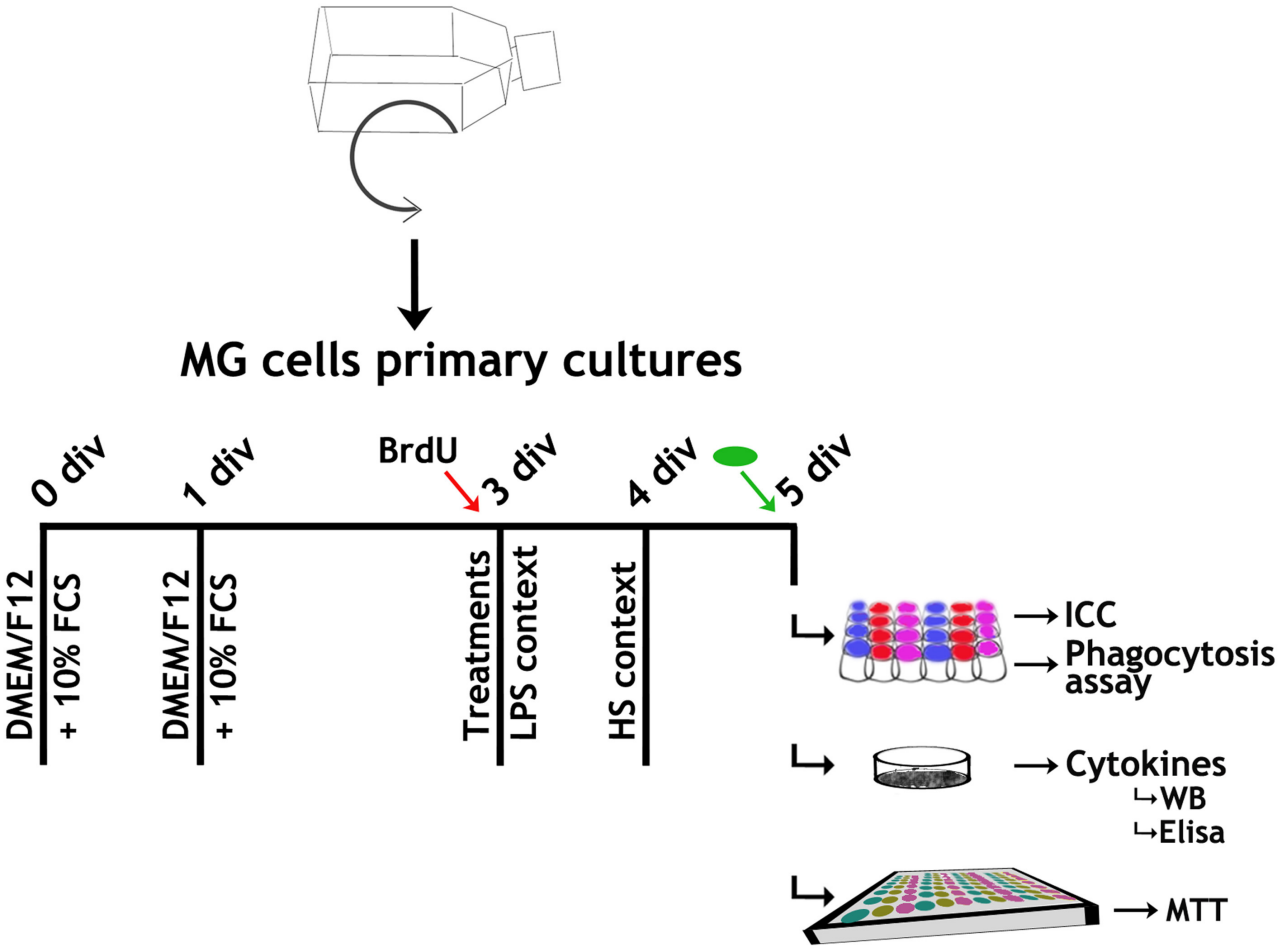
Experimental design. Brains from P0-1 pups born to Wistar rats were dissected and plated on T75 polylysinated flasks. Microglial (MG) cells were harvested by 2 h shaking at 37°C. MG cells were plated either on uncoated coverslips placed in a 24-well plate (7 × 10^3^ cells/well) for ICC and phagocytosis assays, 96-well plate (2 × 10^4^ cells/well) for MTT assays, or 25 mm ø/ Petri dishes (7 × 10^5^ cells/dish) for cytokine quantification, and cultured in Dulbecco's Modified Eagle's medium supplemented with F12 (DMEM/F12) and 10% FCS for 24 h, when medium was changed to remove non-attached cells. At 3 *div*, medium was replaced by the corresponding Stx2 incubation and LPS context applied concurrently with BrdU incorporation (red arrow) where appropriate. At 4 *div*, HS context was applied. At 5 *div*, where applicable, FITC-yeast conjugated incubation (green oval) was performed before fixation. Cultures suitable for ICC and phagocytosis assays were fixed by cold 100% methanol incubation (30 min, −20°C) and washed twice with PBS. On the other hand, for MTT assays and cytokine quantifications, cultures were washed with PBS without fixing.

Total brains of post-natal day (P) 0–1 Wistar rat pups from both sex (8–10 animals/litter) were dissected and plated on T75 polylysinated flasks and cultured in Dulbecco's Modified Eagle's medium (DMEM) supplemented with F12 (DMEM/F12) and 10% fetal calf serum (FCS) for 11–13 days. Following the original protocol (de Vellis and Cole, 2012; Rosato-Siri et al., 2018), MG cells were obtained by differential centrifugation and plated on uncoated surfaces, suitable for the subsequent assay (Figure 1). MG cells culture purity (CD11b positive cells) was checked regarding neuron, oligodendrocytes and astrocytes contamination by NeuN, O4, and GFAP ICC, respectively, rendering 98% pure MG cells cultures (data not shown).

### MG Cell Treatments

MG cells were cultured maintaining DMEM/F12 + 10% FCS and, 48 h after plating, media were changed to DMEM/F12 + 10% FCS (Control) or DMEM/F12 + 10% FCS and the corresponding treatment. Two culture contexts were evaluated: lipopolysaccharide challenge (LPS) (Landoni et al., 2010) and heat shock exposure (HS) (Sugimoto et al., 2014) (Figure 1 and Table 1).

**Table 1:** Experimental groups.

**Culture contexts**	**Control**	**LPS**	**HS**	**LPS + HS**
**Stx2 concentration range**	**Stx2 (ng/ml)**			
	**1**	**10**	**100**	**1,000**
Treatments	Stx2 100 ng/ml W/O	Stx2 100 ng/ml +LPS	Stx2 100 ng/ml +HS	Stx2 100 ng/ml +LPS + HS
	Stx2B 100 ng/ml (W/O)	Stx2B 100 ng/ml +LPS	Stx2B 100 ng/ml +HS	Stx2B 100 ng/ml +LPS +HS

*MG cell behavior was initially evaluated in four culture contexts: Control, LPS, HS, and LPS + HS. In order to evaluate toxin uptake, Stx2 concentration range (1, 10, 100, and 1,000 ng/ml) was assayed. Given that Stx2 100 ng/ml was the concentration condition for which toxin detection was most clearly positive regardless of culture context, effects on toxin uptake were compared at this concentration from here onwards. LPS: 500 ng/ml of lipopolysaccharide was added together with Stx2/Stx2B to culture media. HS: MG cultures were exposed to 42°C for 30 min. W/O: without context. Stx2B was a generous gift from Dr. Mariano Enrique Fernandez-Miyakawa (Instituto de Patobiología, CNIA-INTA)*.

### BrdU Incorporation

BrdU (10 μM) was added concomitantly with treatments (Figure 1, red arrows).

### Immunofluorescence Assay

Immunocytochemistry (ICC) was performed as previously described (Rosato-Siri et al., 2018). Briefly, covers were washed with phosphate buffered saline (PBS), permeabilized (PBS + 0.1% Triton X-100 + 5% FCS) for 3 h and sequentially exposed to primary antibodies (PBS + 0.1% Triton X-100 + 1% FCS) and secondary antibodies (PBS + 0.1% Triton X-100 + 1% FCS) (Table 2). BrdU was incorporated before the ICC protocol by incubated cells in a 2 N HCl solution for 30 min at room temperature and subsequent washes with 100 mM buffer borate, pH 8.5. No immunolabeling was detected when primary antibodies were omitted.

**Table 2:** ICC working antibody dilutions.

**ICC markers**	**Catalog number**	**Dilution**	**Secondary antibodies (1:500)**
Anti-Stx2	^**^	1:500	Anti-Rb Alexa 488
Anti-CD11b	CBL1512 (Millipore)	1:500	Anti-Mo Alexa 647
Anti-Iba1	Ab5076 (Abcam)	1:250	Anti-Gt Cy3
Anti-Gb3	357102 (BioLegend)	1:200	Anti-Mo Alexa 488
Anti-BrdU	11170376001 (Sigma)	1:500	Anti-Mo Alexa 488

** *Anti-Stx2 was a generous gift from Dr. Mariano Enrique Fernandez-Miyakawa. Hoechst dye was added together with the secondary antibody solution. Secondary antibodies were purchased from Jackson ImmunoResearch*.

### Microscopy and Image Analysis

Digital images were acquired either with an epifluorescence Olympus BX50 microscope equipped with a CoolSnap digital camera or with a confocal Olympus FV1000 microscope.

#### Quantification Strategies: FIJI Software Was Used Throughout

Incorporated Stx2 or Iba1 expression: images were subjected to Image <Color <Split Channels, and “Wand tool” was used to select the corresponding marker. “Threshold tool” and Shanbhag or Triangle algorithm, were used to determine fluorescent values expressed as integrated optical density (IOD) average. For Iba1 plot, the control value was subtracted from the treatment ones.

Cytokines in WB membranes: images were converted to 8-bit Image <Type <8-bit. “Rectangle tool” was used to select each band surface. “Wand tool” was used to measure histogram areas under the curve. Values were referred to those of β-actin.

Irregular nuclei: a criterion was used to determine normal and irregular nuclei. Round nuclei were regarded as normal, while all nuclei phenotypes different from this were considered irregular. “Multi-point tool” was used in RGB images to quantify different Hoechst nuclei phenotypes. Values were expressed as percentages.

BrdU^+^ cells or FITC-yeast: RGB images were combined with Image < Color < Merge channels, Hoechst labeling was assigned to the blue channel and BrdU^+^ cells or FITC-yeast were assigned to the green channel. Contrast was established to create clear images. “Multi-point tool” was used to quantify events/field. Values were expressed as percentages.

### MTT Reduction Assay

3-(4,5-dimethyl-thiazol-2-yl)-2,5-diphenyl-tetrazolium bromide (MTT) reduction assay was carried out according to the protocol described by Mosmann (1983). Measurements were expressed as absorbance at 570 nm of each sample relative to the Control group.

### Phagocytosis Assay

MG cells were incubated 15, 30, and 60 min with FITC-yeast conjugated (2.5 × 10^5^ yeast/ml). After incubation, cultures were generously washed with PBS to remove non-phagocytized yeast before fixing (Figure 1, green arrows). Data were expressed either as the number of phagocytized yeast/cell or as a phagocytic index,

$$\begin{array}{l} phagocytic\;index=\frac{number\;of\;phagocytic\;cell\;treatment/field}{number\;of\;phagocytic\;cell\;Control/field} \end{array}$$

### Cytokine Expression Levels

#### Preparation of Lysates From Cell Cultures

The cell suspension was transferred into a microcentrifuge tube, added ice-cold lysis buffer (1 ml per 10^6^ cells, 150 mM sodium chloride, 1.0% NP−40, 0.5% sodium deoxycholate, 0.1% SDS, 50 mM Tris, pH 8.0 and protease inhibitor cocktail) and incubated 15 min at 4°C. The suspension was then centrifuged at 4°C (20 min at 12,000 rpm). The supernatants were placed in a fresh tube and Bradford's method was used to determine protein concentration.

#### Protein Precipitation

The supernatant was mixed with 100% (w/v) trichloroacetic acid (relation 4:1), incubated for 10 min at 4°C and centrifuged for 5 min at 14,000 rpm. The supernatant was then removed and washed three times in cold acetone with 5 min centrifugation at 14,000 rpm each time. Finally, the pellet was dried at 95°C for 5 min and re-suspended on sample buffer 2 × for SDS-PAGE.

#### Western Blot Analysis

Cell extracts (50μg protein) were separated by 12% SDS-PAGE and transferred onto nitrocellulose sheets (GE Life Sciences). After transfer, non-specific binding sites were blocked with 3% BSA in 30 mM Tris, 0.14 M NaCl, 0.1% (v/v), Tween 20, pH 8.0 for 1 h at room temperature with shaking. Specific antibodies were incubated at 4°C overnight (Table 3).

**Table 3:** WB working antibody dilutions.

**WB markers**	**Catalog number**	**Dilution**
Anti-IL1β, clone B122	sc-12742 (Santa Cruz, Biotech.)	1:1,000
Biotin anti-IFNγ	505803 (BioLegend)	1:1,000
Biotin anti-IL10	554465 (BD Biosciences)	1:500
Biotin anti-TGFβ	555053 (BD Biosciences)	1:500
Anti-β-actin	622101 (BioLegend)	1:500

*The corresponding horseradish peroxidase (HRP)-conjugated secondary antibodies were purchased from Jackson ImmunoResearch. Streptavidin-horseradish peroxidase (SAv-HRP) was purchased from BioLegend and used for biotin-tag detection. Loading control: β-actin*.

#### ELISA

The supernatant cytokine levels were measured by ELISA BD OptEIA kit (BD Biosciences, 559603 and 555138) according to the manufacturer's instructions.

### Statistical Analysis

Data (*n* = 9, independent cultures) were subjected to non-parametric tests suitable for normally distributed populations. Statistical analysis was performed using one-way ANOVA followed by different post-tests (GraphPad Prism, GraphPad Software Inc.). The corresponding replicates for consistency, *p*-values and post-tests are provided in each figure legend.

## Results

### MG Uptake of Stx2 Depends on Toxin Concentration and Culture Contexts

**Figure 2:**
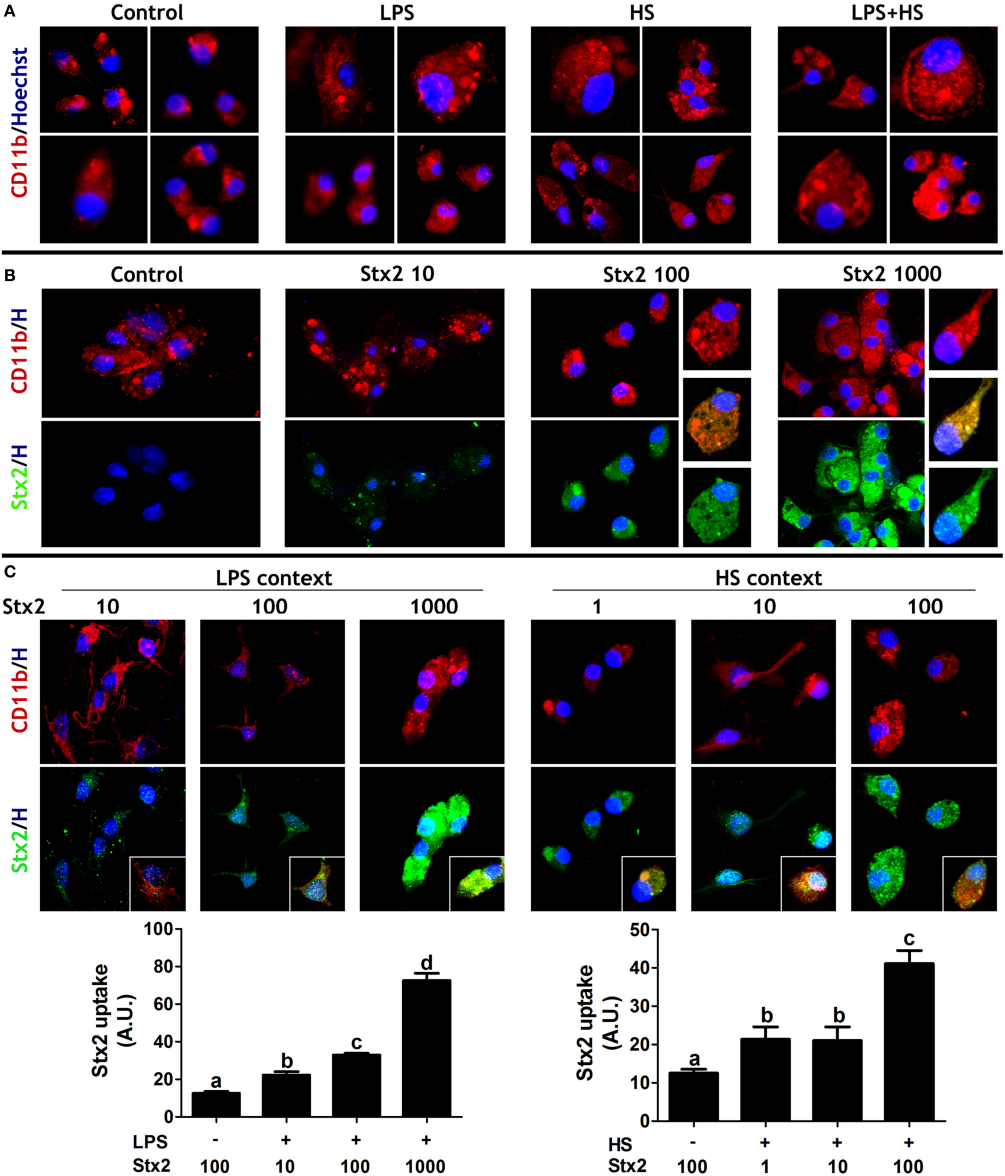
MG cell culture contexts and Stx2 uptake. **(A)** MG cells behavior was evaluated in different contexts: Control, LPS, HS, or LPS + HS. **(B)** Stx2 uptake was evaluated upon different toxin concentrations (1, 10, 100, or 1,000 ng/ml). **(C)** Stx2 uptake was also evaluated and quantified in LPS and HS contexts. Significant differences are indicated by different letters (a–d, *p* < 0.05). Bonferroni's *post-hoc* test *n* = 5, replicates = 3. CD11b (red) and Stx2 (green) ICC showed MG cells specific uptake of Stx2. Insets: Higher magnifications of double positive cells (merge images).

MG cell behavior was initially evaluated in four culture contexts: Control, LPS, HS, and LPS + HS. CD11b detection, a marker for all types of sensing and responsive MG cell phenotypes, showed that neither viability nor survival were affected in 98% pure MG cell cultures in any context assayed (Figure 2A). MG cells were incubated in a Stx2 concentration range (1, 10, 100, and 1,000 ng/ml) in order to evaluate toxin uptake. Stx2 immunodetection was clearly positive in CD11b^+^ cells incubated with 100 and 1,000 ng/ml (Figure 2B) but weakly positive and negative when MG cells were incubated with 10 ng/ml and 1 ng/ml (data not shown), respectively. In addition, the Stx2 concentration range was evaluated in two culture contexts. Interestingly, Stx2 uptake at 10 ng/ml turned out to be positive in LPS and HS as compared to Stx2 incubation without (W/O) context, while Stx2 uptake at 1 ng/ml was only positive in HS. Stx2 uptake at 100 ng/ml and 1,000 ng/ml increased in both contexts tested as compared to incubations W/O context (Figure 2C). Given that Stx2 100 ng/ml was the concentration condition for which toxin detection was most clearly positive regardless of culture context, effects on toxin uptake were compared at this concentration from here onwards.

### MG Cell Activation Increases With Stx2 and Challenged Culture Contexts

**Figure 3:**
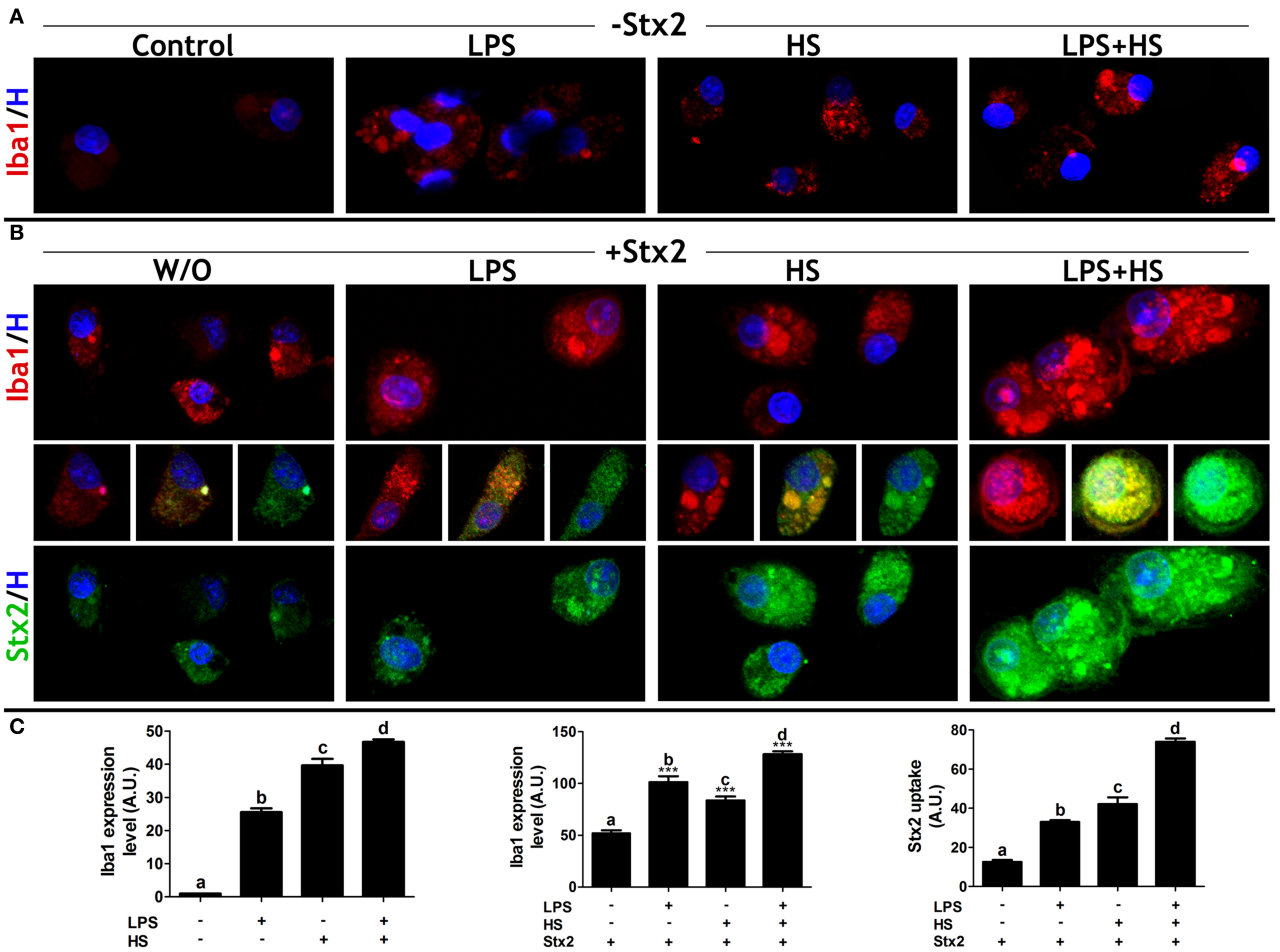
MG responsiveness and Stx2 uptake. **(A)** Iba1 ICC in MG cells cultured in different contexts. **(B)** Iba1 expression level (upper panel) and Stx2 uptake (lower panel) by ICC in MG cells exposed to Stx2 100 ng/ml. Middle images: merged Iba1, Stx2, and Hoechst labels. **(C)** Iba1 expression level and Stx2 uptake quantifications. Significant differences are indicated in comparison with Control (a–d, *p* < 0.05) or in comparison with Stx2 W/O context (***p < 0,001). Bonferroni's *post-hoc* test; *n* = 5; replicates = 3.

Ionized calcium-binding adaptor protein1 (Iba1) is an actin-binding protein constitutively and specifically expressed in all MG cells while activated MG cells are associated with increased Iba1 expression (Imai et al., 1996; Sasaki et al., 2001). MG cell activation status was determined by Iba1 immunofluorescence in the contexts previously described. Iba1 expression levels were increased in both LPS and HS, taken together or separately in comparison with Control (Figures 3A,C). MG cell activation was also evaluated upon Stx2 at 100 ng/ml incubation. Toxin *per se* produced an increase in Iba1 expression levels in comparison with Control. This increase was significantly more evident when cultures were exposed to Stx2 and LPS or HS and showed a synergistic effect when LPS + HS were applied (Figure 3B, upper panel and Figure 3C). In addition, Stx2 uptake quantification at 100 ng/ml verified the qualitative increase observed in culture contexts (Figure 2B). Interestingly, toxin uptake was markedly increased in LPS + HS (Figure 3B, lower panel and Figure 3C).

### MG Cell Metabolic Activity Increases With Stx2 and LPS-Challenged Culture Contexts

**Figure 4:**
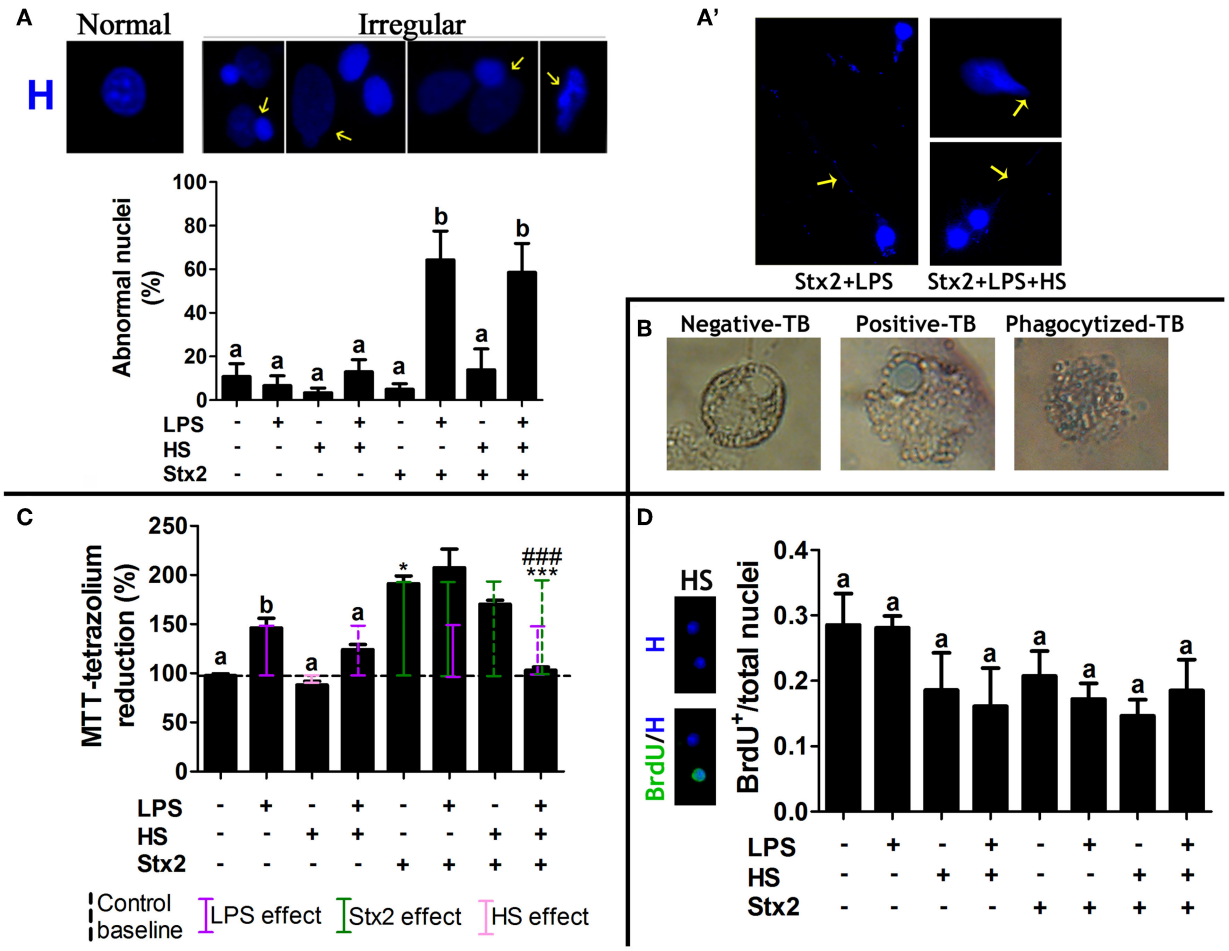
MG cell cytological and metabolic features. **(A)** Quantification of irregular nuclei phenotype (yellow arrows). Significant differences are indicated by different letters in comparison with Control (*p* < 0.05). Bonferroni's *post-hoc* test. *n* = 5; replicates = 5. **(A′)** Extra-cellular trap-like structure (ET-LS) was only observed in Stx2 + LPS and Stx2 + LPS + HS. **(B)** TB staining (1:1 dilution of the cell suspension and a 0.4% Trypan Blue solution). **(C)** MTT assay. Significant differences are indicated as follows: a and b, between context effects and Control (*p* < 0.05); * between Stx2 W/O context and Control (*p* < 0.05); *** between Stx2 + LPS + HS and all the other Stx2 incubations (*p* < 0.001), and ^###^ between Stx2 + LPS + HS and Stx2 + LPS (*p* < 0.001). Newman-Keuls's *post-hoc* test; *n* = 5; replicates = 12. **(D)** BrdU-positive nuclei quantification showed no significant differences across contexts; Bonferroni's *post-hoc* test; *n* = 4; replicates = 3.

To further describe MG cell behavior, cytological, and metabolic features were evaluated concurrently with culture conditions. Nuclear morphology was typified considering shape, size, cellular location, and staining intensity. Normal nuclei were round with a defined edge, often centrally located and with homogenous Hoechst fluorescence staining. In contrast, irregular nuclei were larger and not round, exhibiting peripheral location and diffuse Hoechst staining (Figure 4A, yellow arrows). Only Stx2 + LPS and Stx2 + LPS + HS showed an increase in the mean number of irregular nuclei (Figure 4A), in addition to a Höechst-positive configuration resembling extra cellular trap-like structures (ET-LS) (Figure 4A′).

The Trypan blue (TB) viability assay was performed to evaluate cell death. Three different situations regarding TB staining were observed, i.e., negative and positive nuclear staining, and a third staining corresponding to phagocytized TB (Figure 4B); the latter, would be recorded as a positive stain if TB intracellular localization had not been considered. Phase contrast images showed no significant differences of TB-positive nuclear staining across groups (data not shown). Unlike HS, LPS alone increased the MTT score (Figure 4C, solid violet line) as compared to Control (Figure 4C, dashed black line). Stx2 also significantly increased the MTT score over Control (Figure 4C, solid green line). Interestingly, opposite effects were observed when Stx2 exposure was compared either in LPS or HS context. Although no significant, a modest increase was observed in Stx2 + LPS condition while a decrease was detected in Stx2 + HS one. A sharper synergic effect was observed in Stx2 + LPS and Stx2 + HS as compared to LPS and HS, respectively. Surprisingly, Stx2 + LPS + HS showed an attenuated effect (Figure 4C, dashed violet and green lines). Finally, BrdU^+^ nuclei quantification corroborated that MTT findings were associated with metabolic activity (Gerlier and Thomasset, 1986) rather than cell viability (Figure 4D).

### Subcellular Presence and Distribution of Stx2 Receptor Gb3 Depend on Culture Contexts

**Figure 5:**
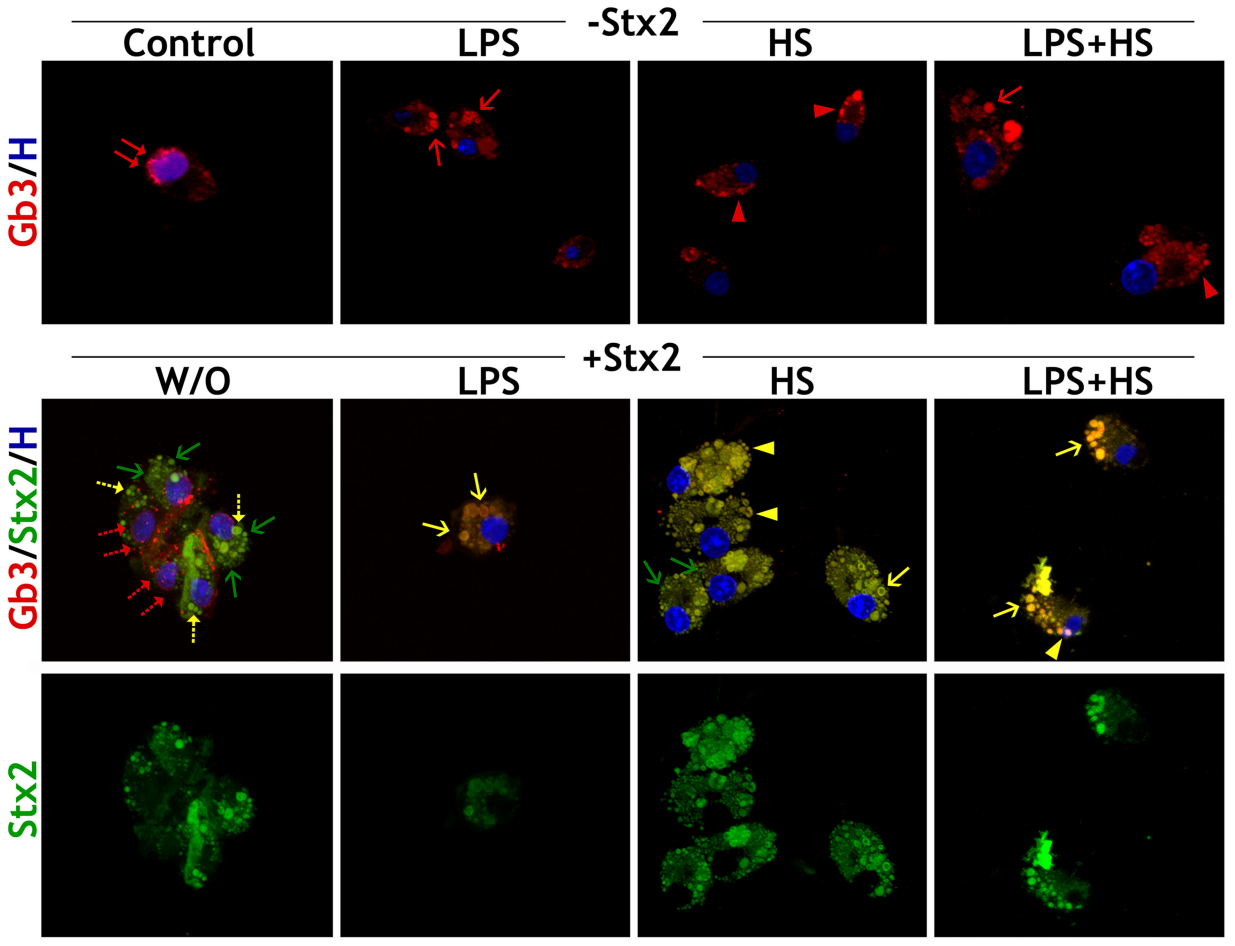
MG cell Gb3 distribution pattern. Stx2 and Gb3 detection by ICC in different culture contexts (LPS, HS, LPS + HS), Control and Stx2 100 ng/ml incubations W/O and with contexts. Merge of Gb3/Stx2/Hoechst labeling. Double red arrows: perinuclear location of Gb3; single red arrows: vesicle location of Gb3; red arrowheads: membrane location of Gb3; single green arrows: toxin vesicles without Gb3; single yellow arrows: vesicle location of Stx2 + Gb3; yellow arrowheads: membrane location of Stx2 + Gb3. Dotted-arrows represent specific distribution reductions. *n* = 4, replicates = 3.

Gb3 presence and subcellular distribution were determined by ICC. Depending on MG cell culture context, Gb3 expression showed three clearly distinguishable distribution patterns: perinuclear in Control, vesicular in LPS and on the cell membrane in HS (Figure 5, double red arrows, single red arrow, and red arrowhead, respectively). In turn, LPS + HS showed Gb3 vesicular and cell surface coexistence (Figure 5, single red arrow and red arrowhead).

MG cell incubation with Stx2 alone rendered double immunofluorescence, which indicates that there is a Stx2 uptake that seems to be independent of Gb3 binding (Figure 5, single green arrow) along with a Gb3-dependent uptake depicted by a few double immunofluorescence vesicles (Figure 5, single yellow dotted-arrow). In addition, Gb3 perinuclear distribution was reduced upon Stx2 exposure (Figure 5, double red dotted-arrow) in comparison with Control condition. In contrast, the Gb3 canonical pathway underlay most of the Stx2 uptake in LPS, HS, and LPS + HS, as double immunolabeling matched the specific pattern characterizing each context: only large vesicular arrangement for LPS condition, clusters localized in nearby cell surface for HS condition and a combination of both patterns when LPS and HS were applied (Figure 5, single yellow arrow and arrowheads).

### MG Cells Behave Similarly Upon Stx2 and Stx2B Exposure

**Figure 6:**
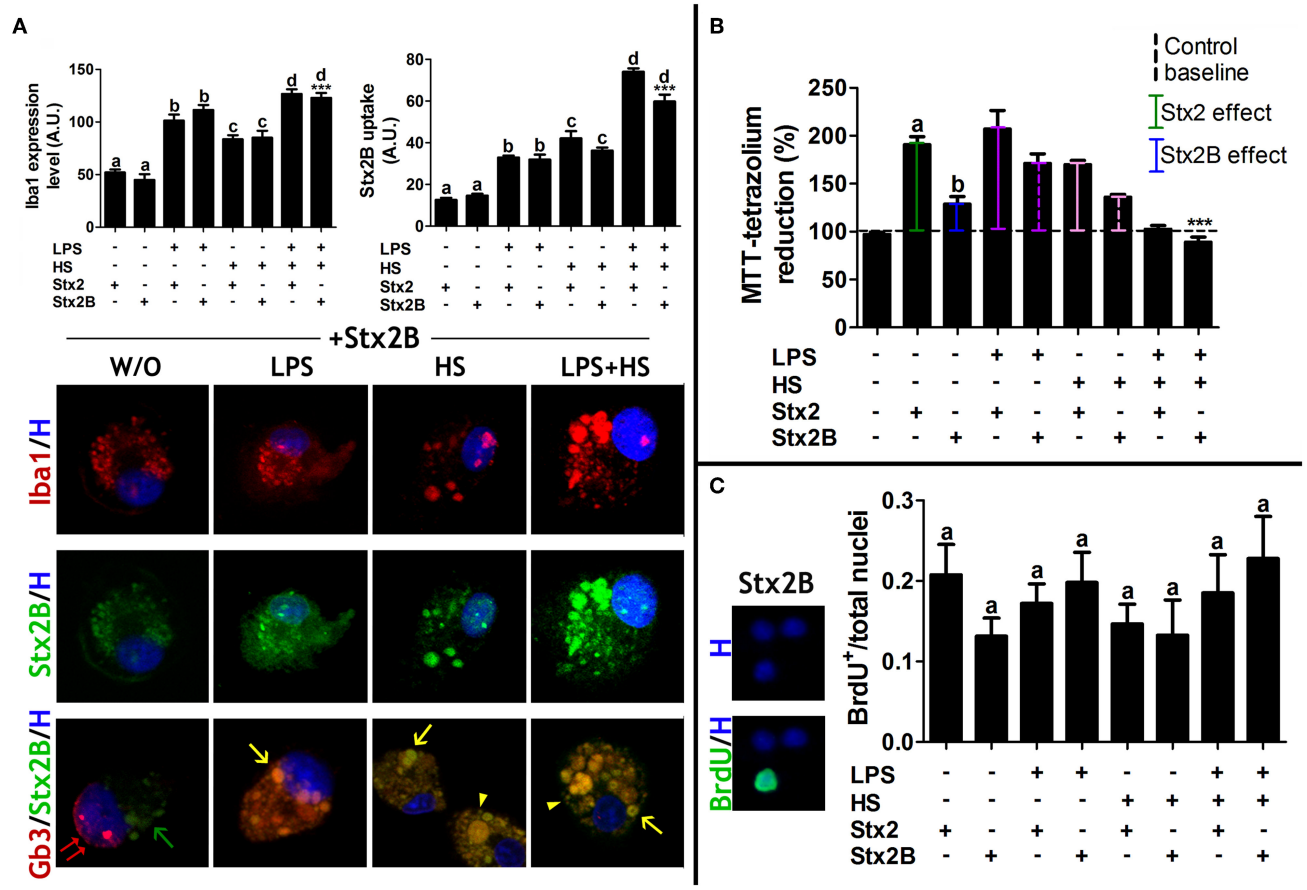
MG cell responsiveness and Stx2B uptake. **(A)** Iba1 expression level and Stx2B uptake quantifications in MG cell cultures. Lower images: merge of Gb3/Stx2B/Hoechst labeling. Double red arrows: perinuclear location of Gb3; single green arrows: toxin vesicles without Gb3; single yellow arrows: vesicle location of Stx2B + Gb3; yellow arrowheads: membrane location of Stx2 + Gb3. Significant differences are indicated by different letters comparing Stx2 and Stx2B in the same context (a: W/O context, b: LPS, c: HS, d: LPS + HS; *p* < 0.05). Significant differences in comparison with Stx2B, Stx2B + LPS and Stx2B + HS (***p < 0.001). Bonferroni's *post-hoc* test; *n* = 4; replicates = 4. **(B)** MTT assay. Significant differences between Stx2 and Stx2B (a and b, *p* < 0.05). Newman-Keuls's *post-hoc* test; *n* = 5; replicates = 12. **(C)** BrdU-positive nuclei quantification showed no significant differences across contexts. Bonferroni's *post-hoc* test; *n* = 4; replicates = 3.

As part of the Stx family, Stx2 has an AB5 structure in which a single subunit A is associated with five subunits B. Subunit A represents the catalytic activity while the B-pentamer (Stx2B) is the binding site of the Stx2-Gb3 receptor (Fraser et al., 2004). Recombinant Stx2B (Parma et al., 2011) was used to compare its biological effects on MG cells with the Stx2 one. Iba1 expression levels equally increased in both Stx2- and Stx2B-incubated MG cells, with or W/O context. Once again, MG cell responsiveness was significantly more evident when cultures were exposed to Stx2B and LPS, HS, or LPS + HS (Figure 6A, left panel). Environmental cues tuned up Stx2B uptake in the same manner as for Stx2; accordingly Stx2B uptake quantification showed significant increments when cultures were exposed to Stx2B and LPS, HS, or LPS + HS contexts. Interestingly, no significant differences were observed comparing Stx2 and Stx2B in the same context (Figure 6A, right panel). Gb3 distribution pattern in the presence of Stx2B was similar to that observed in the presence of Stx2, both with and W/O context conditions (Figure 6A, lower panel). However, Stx2B MTT score was significantly lower than that of Stx2 incubation W/O context. In addition, when MG cells were incubated with Stx2B in LPS and HS, MTT values showed a diminishing tendency as compared to Stx2 incubation in the same contexts, reaching a significant decrease in LPS + HS (Figure 6B). Finally, BrdU incorporation (Figure 6C) and Trypan blue assays (data not shown) showed non-significant differences between groups.

### MG Cell Phagocytic Profile Is Independent of Stx2 Catalytic Subunit

**Figure 7:**
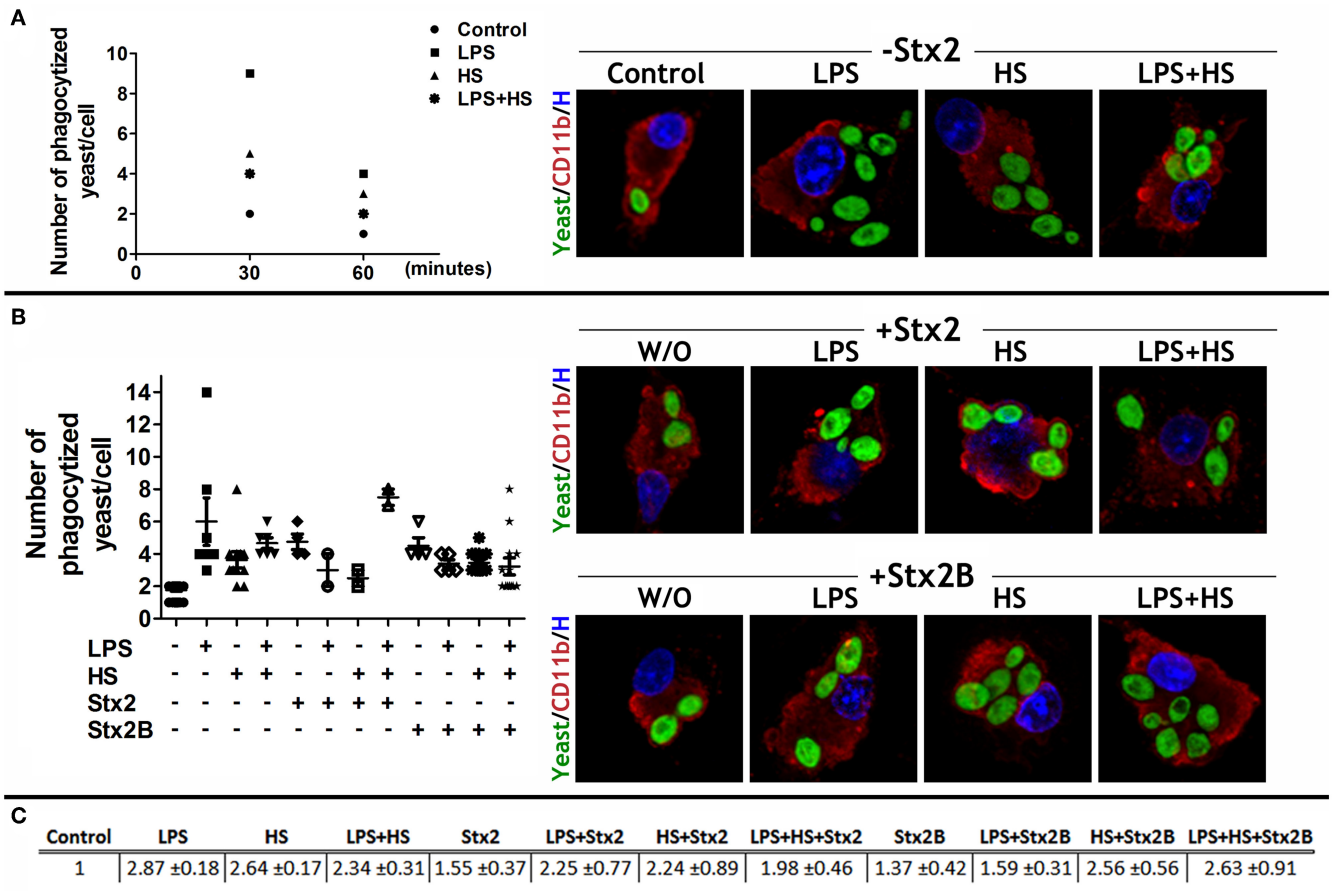
MG cell phagocytic profile. **(A)** The phagocytic kinetics was determined in MG cells cultured in LPS, HS, and LPS + HS contexts by incubating them with FITC-yeast conjugated for 15, 30, and 60 min. Fifteen-min incubation was not sufficient to bring about significant differences across treatments but 30 min incubation displayed stark differences. **(B)** MG cells challenged with Stx2 or Stx2B and specific culture context were incubated for 30 min with FITC-yeast. Data are expressed either as the number of phagocytized yeast/cell or **(C)** as a phagocytic index (*p* < 0.05). Bonferroni's *post-hoc* test; *n* = 4; replicates = 4.

MG cell activity was described through a phagocytosis assay. MG cells were incubated 15, 30, and 60 min with FITC-yeast conjugated in order to determine the phagocytic kinetics and the saturation point. Comparing Control and context conditions, differences in the maximum phagocytized yeast were most evident after 30 min incubation (Figure 7A). Following 30 min incubation, the frequency distribution in the number of phagocytized yeast/cell showed that all treatments increased in comparison with Control, being LPS condition the higher one. In addition, both Stx2 and Stx2B incubations increased the number of phagocytized yeast/cell in comparison with Control group but showed similar results to each other (Figure 7B). The phagocytic index showed that toxin incubation alone seem to have no profound effect on phagocytic efficiency. Remarkably, context challenges strongly tuned up toxin, holo, and B-subunit, effects by increasing MG cell phagocytic efficiency, as more MG cells/field were actively engaged in the process. Interestingly, the increase triggered by LPS on Stx2 effect was attenuated when HS was combined with them (Figure 7C).

### MG Cell Pro-Inflammatory Profile Is Positively Dependent on Stx2 Incubation and Challenged Culture Contexts

**Figure 8:**
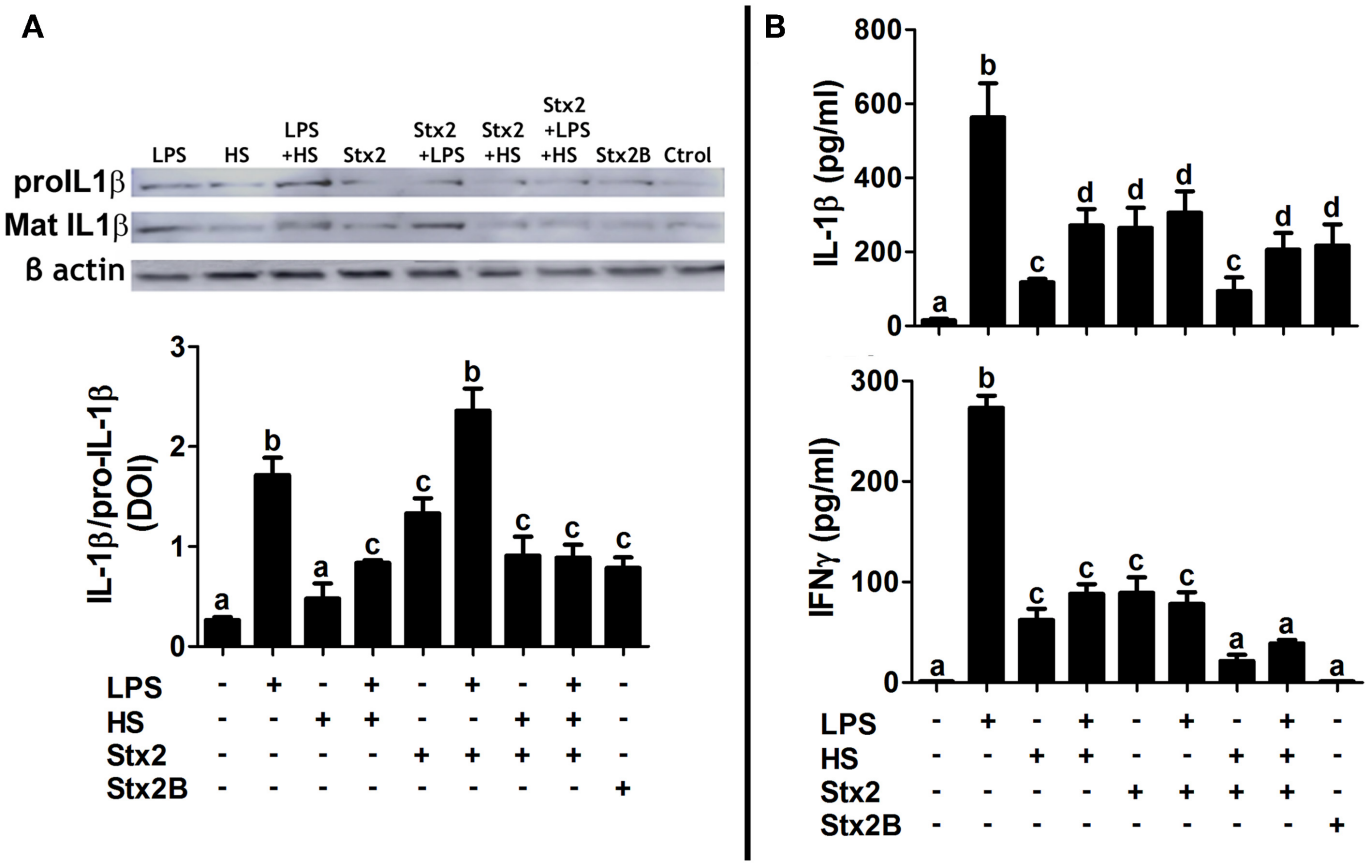
MG cell cytokine profile. **(A)** IL1β expression levels quantified by WB on cellular fractions. Pro IL1β: IL1β not active. Mat IL1β: cleaved and active IL1β. Data are expressed as mean IOD ± SEM relative to β-actin detection. **(B)** ELISA on culture supernatants to quantify IL1β and IFNγ release. Significant differences are expressed as different letters (a–d, *p* < 0.01). Dunnett's multiple comparison *post-hoc* test; *n* = 3; replicates = 2.

Cytokine profiles were evaluated either by WB, to detect cytokine expression levels on cellular fractions (Figure 8A), or by ELISA, to determine cytokine release on culture supernatants (Figure 8B). As expected, the LPS context showed a pro-inflammatory profile, increasing IL1β and IFNγ synthesis and release. Stx2 and Stx2B incubation W/O context also increased IL1β synthesis and release, whereas only Stx2 increased IFNγ release. IFNγ synthesis was undetectable under Stx2 or Stx2B exposure (data not shown). Interestingly, Stx2 + LPS exhibited a synergic effect on IL1β expression levels, but both IL1β and IFNγ release showed no significant differences with Stx2 alone. HS increased IL1β and IFNγ release in comparison with Control, although the cytokine pro-inflammatory profiles triggered by LPS and Stx2 exposures were attenuated when HS was combined with them. None of the treatments tested showed detectable levels of anti-inflammatory cytokines IL10 and IL6 (data not shown).

## Discussion

MG cells, with a mesodermal/mesenchymal developmental origin, are considered CNS resident macrophages. MG cells constitute the most reliable environmental sensors in the brain and “resting” MG cells status seems somewhat a misnomer since they stand for a continuous sensing state. Upon the detection of a variety of CNS dysfunctions, MG cells undergo a complex, multistage activation process that converts them into an activated status (Kettenmann et al., 2011). Pathogenesis of CNS during the acute phase of HUS is known to correlate with brain-blood-barrier disruption, neuronal disturbances and astrogliosis (Pinto et al., 2017), although less is known so far about MG cell behavior and their role in the events observed in these CNS complications.

The present work explored the capacity of MG cells to uptake different concentrations of Stx2 and Stx2B in different contexts: HS, LPS, and LPS + HS. In addition, the Gb3 canonical pathway for holotoxin uptake was analyzed comparing the biological response of MG cells to either Stx2 or Stx2B. Finally, MG cell activation, metabolic activity, phagocytic profile, and cytokine release were further analyzed in the different contexts.

MG cell sensitivity, described by intracellular Stx2 quantification, was much higher in HS than in LPS. Moreover, MG cells were so sensitive in HS that low toxin concentrations brought about Stx2 uptake. Interestingly, MG cell responsiveness depicted by activated MG marker Iba1 expression was increased in the three contexts evaluated upon Stx2 exposure. As reflected by the expression of Iba1, Stx2, and also Stx2B succeeded in activating MG cells in all contexts. However, this activated state only coincided with increased metabolic activity rather than proliferative status when these cells were treated with Stx2 in LPS, but not when MG cells where incubated with Stx2B in the same context. In addition, pro-inflammatory cytokine expression levels were equally increased by Stx2 and Stx2B incubations, although their release seemed to be dependent on the holotoxin effect. Therefore, it can be inferred that metabolic activity and cytokine release depend on the holotoxin, where the catalytic and binding subunit—Stx2B—are integrated. On the other hand, our results confirm that B-pentamer is responsible for Stx2 uptake in MG cells, noting that environmental cues modified Stx2B effect in the same manner as for Stx2.

In being reactive to Stx2, MG cells could play a pivotal role in inflammatory mechanisms underlying cellular damage associated with neurological dysfunctions. In fact, previous work has demonstrated the role of MG cells in system homeostasis through a physiological balance of pro-inflammatory and anti-inflammatory profiles (Sirerol-Piquer et al., 2019); thus, a shift toward a pro-inflammatory profile correlates with physiological and system damage. Under the present acute experimental conditions, toxin incubation triggered a pro-inflammatory profile in terms of IL1β and IFNγ synthesis and release, with a sharper effect when the catalytic subunit was present. In addition, environmental cues tuned up Stx2 effect: on the one hand, LPS context increased pro-inflammatory cytokine synthesis. On the other hand, HS treatment softened MG cells activation, even though a pro-inflammatory profile remains. It is worth mentioning that the two techniques used to determine cytokine synthesis and release, WB and ELISA, respectively, show different threshold sensitivity, the latter allowing for easy detection of cytokines on supernatants as compared to the corresponding protein product. Bearing in mind that IFNγ concentrations were under 300pg/ml, it is reasonable to infer that its synthesis was undetectable by WB.

The present work also demonstrates, for the first time, that MG cells exhibit both a Gb3-independent and Gb3 canonical pathway for Stx2 uptake. Our results seem to be in line with Johannes review (Johannes, 2017), which reported toxin clustering on the plasma membrane as well as receptor binding and intracellular trafficking by endocytic carriers for the Gb3 canonical pathway. Stx2 has been seen associated with clathrin-coated pits (Sandvig et al., 1989) but, even though clathrin function was proficiently inhibited, a 75% of Shiga toxin uptake remained intact (Lauvrak et al., 2004). In addition, the present results agree and elaborate on those obtained by Griener et al. (2007), where human neutrophils exhibited Gb3-independent Stx2 uptake, murine neutrophils showed the classical Gb3-dependent Stx2 uptake, and MG cells showed both Stx2 uptake pathways. A recent work by Brigotti et al. (2018) has beautifully confirmed that toll-like receptor 4 (TLR4) is mainly responsible for Stx2 interaction on neutrophil membrane. TLRs are receptors expressed by cells with an innate immune profile, such as MG cells, which are stimulated by pathogen-associated molecular patterns (PAMPs), including LPS. Most importantly, LPS/TLR4 signaling triggers the expression of pro-inflammatory cytokines (Lu et al., 2008). This pathway has been proved that MG cell activation underlies neuro-degenerative mechanisms (Jiang et al., 2016) and even linked CNS effects and systemic inflammation (Zhou et al., 2019). Bearing this in mind, we observed that Gb3-independent Stx2 uptake was no longer detectable in LPS context.

MG cells are specialists in phagocytosis, and the current work shows an increase in their phagocytic efficiency upon toxin exposure. Moreover, as Stx2B also yielded phagocytic properties, phagocytosis appeared to be independent of the Stx2A catalytic subunit. Environmental conditions, by themselves, have positively changed MG cells phagocytic performance and also emphasized toxin effect, Stx2 and Stx2B, since MG cell engagement to a phagocytic profile was increased when LPS was applied. On the contrary, HS treatment attenuated MG cells phagocytic profile when combine with LPS. Phagocytic status is not only an activated MG cell feature but also a spillover effect to regulate system homeostasis, conferring MG cells a neuroprotective function through dying cells clearance, preventing pro-inflammatory and neurotoxic molecules events (Hamzei Taj et al., 2016; Wolf et al., 2017; Janda et al., 2018). It has been showed that MG cells phagocytosis impairment worsens Alzheimer Disease pathology (Noda and Suzumura, 2012). In this regards, previous results have revealed cellular deleterious effects by STEC and LPS actions, the presence of Stx2 in abnormal-phenotype neurons and neuronal damage which correlate with MG cells reactivity (Goldstein et al., 2007; Pinto et al., 2018; Berdasco et al., 2019). Former and present results build up one another showing that phagocytic MG cells correlated either with MG cells clearance function of Stx2-induced debris or Stx2 direct effect.

Several report shave established that cell nuclei can display diverse morphologies which correlate with functions and specific cell processes, for example, chromatin remodeling correlating with neutrophil activation steps (Carvalho et al., 2015). Interestingly, nuclear reshaping has been observed in macrophages in response not only to environmental conditions but also to *Bacillus anthracis* edema toxin (Trescos et al., 2015), although macrophage functionality was unaltered. Mosser and Edwards (2008) established a link between nucleus remodeling with an increase cellular plasticity to change between roles since readily deformable nucleus facilitates transcriptional regulation (Skinner and Johnson, 2017). To our knowledge, no similar data have been reported thus far on MG cells. Worth highlighting, irregular nuclei morphology in the current work was observed only when MG cells were incubated with Stx2 in challenged culture conditions.

ETs have been recently recognized as a defense mechanism in several types of innate immune cells and have been described in eosinophils (Yousefi et al., 2012; Geering et al., 2013), neutrophil—NETs (Clark et al., 2007; Yipp et al., 2012) and macrophages—METs (Liu et al., 2014; Kalsum et al., 2017). An intriguing discovery was that both NETs and METs formation overlapped with intact functional cells, hence called vital ETs (Yousefi et al., 2009), which gives ETs a protective rather than a harmful role (Malachowa et al., 2016). In this scenario, ET-LS observed in MG cells upon specific STEC cues demonstrated the feasibility of this particular cell behavior occurrence in MG cells, as far as we know, described for the first time. Last but not least, and even if we are well aware that this cytological observation should be complemented by additional assessments in order to determine ET-LS molecular nature, our data provide novel insights into the matter and thus worth digging into.

In the light of the events observed in this work, it is easily conceivable that Stx2, LPS, and HS effects on MG cell behavior drive STEC consequences in the CNS. Worth pointing out, that Stx2 incubation does not only reflect a physiological feature of STEC infection but also a baseline over the course of the disease when compared to LPS, HS, and LPS + HS. LPS and HS challenges were used to mimic inflammatory reaction and fever, respectively, being the latter a pathophysiological feature which commonly occurs during HUS-associated encephalopathy. Shimizu et al. (2012) have reported high levels of the endogenous pyrogen TNFα in patients with acute encephalopathy during the HUS phase but physiological ones in the absence of encephalopathy during the HUS phase. In addition, the inflammatory profile triggered by STEC in the gut and then replicated in the CNS by MG cells has been mimicked through the LPS context. On early stages of STEC infection, Stx2 had a direct effect on MG cells, as they were capable of toxin uptake. Stx2 itself, increased MG cells responsiveness, metabolic activity, and pro-inflammatory profile. Later on the physiopathology, environmental cues, particularly LPS challenged, increased both MG cells sensitivity, and responsiveness together with an increase of metabolic activity and phagocytic and pro-inflammatory profiles, over and above Stx2 effect. In addition, upon specific STEC cues, MG cells seems to develop nucleus remodeling and ETs like structures, which could be associated with a plastic and adaptive behavior. As HUS phase moves forward, and features gathered, MG cells behavior exhibited some interesting features attributable only to HS context—fever. In this regards, MG cells increased their sensitivity, as they responded even to low toxin concentrations. This observation correlates with HS effect on Gb3 pattern distribution: by changing Gb3 perinuclear distribution to a clustering localization in nearby the cell surface, even in Stx2 absence, the receptor availability was increased and consequently, cell competence to toxin uptake too. In addition, MG cells metabolic activity and pro-inflammatory profile were attenuated. More important, MG cell phagocytosis was also softened. This fact might well be read as an impairment in MG cells capacity to restore system homeostasis, leading toward HUS-associated encephalopathy.

These findings underscore MG cells pivotal role in inflammatory processes observed in clinical HUS encephalopathies and make inroads into the development of future therapeutic strategies which deserve further investigation.

## Data Availability Statement

The raw data supporting the conclusions of this manuscript will be made available by the authors, without undue reservation, to any qualified researcher.

## Ethics Statement

The animal study was reviewed and approved by Institutional Review Board at Buenos Aires University Council of Animal Care and in accordance with the National Institutes of Health Guide for the Care and Use of Laboratory Animals. Animal protocols were approved by the School of Medicine Committee on Ethics and Animal Research (CICUAL Number: 046/2017).

## Author's Note

Hemolytic Uremic Syndrome is an illness caused by bacterium enterohemhorragic *Escherichia coli*, orally acquired by the ingestion of contaminated food or water or direct transmission. Once inside the gut, this bacterium releases a toxin called Shiga, which is responsible for tissue bleeding and renal failure. Shiga toxin also produces encephalopathies, but little is known about how it leads to neuronal death and whether other brain cells participate in the process. For these reasons, we decided to explore Shiga toxin effects on a particular cell type called microglia. Although less famous than neurons, microglial cells have a crucial role in inflammatory processes inside the brain and are thus probably responsible for the severity of encephalopathies. Our experiments are *in vitro*, which means that we started from a whole rat brain to finally isolate microglial cells and maintain them in a specific culture medium for several days. We then modified culture medium, by adding Shiga toxin for example, and observed that microglial cells have a specific path to incorporate the toxin. Once this happens, these cells increase their metabolism and phagocytic capacity, thus favoring inflammation. These findings make novel inroads in the development of therapeutic strategies with a focus on microglial cells.

## Author Contributions

CB, JG, and MR-S conceived the experiments. CB and MR-S designed and performed the experiments. MD performed the cytokine expression levels assays. CB, JG, and MR-S analyzed the data. MR-S and JG wrote the paper.

### Conflict of Interest

The authors declare that the research was conducted in the absence of any commercial or financial relationships that could be construed as a potential conflict of interest.
